# Human scaphoid non‐unions exhibit increased osteoclast activity compared to adjacent cancellous bone

**DOI:** 10.1111/jcmm.12677

**Published:** 2015-09-28

**Authors:** Jessica Schira, Matthias Schulte, Carmen Döbele, Christoph Wallner, Stephanie Abraham, Adrien Daigeler, Ulrich Kneser, Marcus Lehnhardt, Björn Behr

**Affiliations:** ^1^ Department of Plastic SurgeryBG University Hospital BergmannsheilRuhr University BochumBochumGermany; ^2^ Department of Plastic SurgeryBG Trauma Hospital LudwigshafenUniversity of HeidelbergLudwigshafenGermany

**Keywords:** non‐union, osteoclasts, scaphoid, *RANKL*, *WNT5A*

## Abstract

Scaphoid bones have a high prevalence for non‐union. Even with adequate treatment, bone regeneration may not occur in certain instances. Although this condition is well described, the molecular pathology of scaphoid non‐unions is still poorly defined. In this study, gene expression of osteogenic and angiogenic growth and transcription factors as well as inflammatory mediators were analysed in human scaphoid non‐unions and intraindividually compared to adjacent autologous cancellous bone from the distal radius. In addition, histology and immunohistochemical stainings were performed to verify qRT‐PCR data. Gene expression analysis revealed a significant up‐regulation of *RANKL, ALP, CYCLIN D1, MMP‐13, OPG, NFATc1, TGF‐*β and *WNT5A* in scaphoid non‐unions. Interestingly, *RANKL* and *NFATc1*, both markers for osteoclastogenesis, were significantly induced in non‐unions. Moreover, *WNT5A* was highly up‐regulated in all non‐union samples. TRAP staining confirmed the observation of induced osteoclastogenesis in non‐unions. With respect to genes related to osteogenesis, alkaline phosphatase was significantly up‐regulated in scaphoid non‐unions. No differences were detectable for other osteogenic genes such as *RUNX‐*2 or *BMP‐*2. Importantly, we did not detect differences in angiogenesis between scaphoid non‐unions and controls in both gene expression and immunohistochemistry. Summarized, our data indicate increased osteoclast activity in scaphoid non‐unions possibly as a result of the alterations in *RANKL, TGF‐*β and *WNT5A* expression levels. These data increase our understanding for the reduced bone regeneration capacity present in scaphoid non‐unions and may translate into the identification of new therapeutic targets to avoid secondary damages and prevent occurrence of non‐unions to scaphoid bones.

## Introduction

Bone fracture healing is typically completed 6–8 weeks after the initial injury without scar formation. Certain circumstances could result in delayed fracture healing or non‐unions which lead to pain and arthritis. Scaphoid bones have by far the highest incidence of fractures among all carpal bones and show a 90–95% union rate. However, those fractures with dislocations greater than 1 mm are associated with a 55% incidence of non‐union 1. In general, non‐unions may result from the instability of the fracture, disrupted vascularity, loss of bone and cyst formation. However, factors and molecular mechanisms that lead to failure of bone regeneration are not well defined 2. Blood supply in scaphoids depends on distal branches of the radial artery, which could result in interrupted blood supply of the proximal scaphoid pole and avascular necrosis after fracture. Non‐unions of the scaphoid are predominantly atrophic, which are historically defined by hypovascularization and little callus formation around a non‐mineralized fibrous tissue‐filled fracture gap 3. Treatment of atrophic non‐unions is difficult and often includes three steps: resection of scar tissue, grafting of autologous bone and internal fixation for mechanical stability.

Bone is a dynamic organ with tightly regulated continuous bone remodelling. Differentiation of bone resorbing osteoclasts (OCs) which share several regulatory molecules with immune cells is mainly regulated by tumour necrosis factor (TNF) superfamily member receptor activator of nuclear factor‐κB ligand (RANKL, encoded by the *Tnfsf11* gene) 4, normally expressed by osteoblasts (OBs) and stromal cells, through binding to its receptor RANK (encoded by the *Tnfrsf11a* gene) 5 and the RANKL antagonist osteoprotegerin (OPG encoded by the *Tnfrsf11b* gene) 6, 7. RANK activation results in translocation of c‐Fos into the nucleus forming dimers with the AP‐1 transcription factor complex which together with nuclear factor of activated T cells c (NFATc) activates OC‐specific genes 8.

Osteoclast differentiation is further controlled by the presence of macrophage/colony‐stimulating factor (M‐CSF) 9, which could induce RANK expression 10 followed by the activation of nuclear factor‐κB (NF‐κB) and AP‐1.

Tumour necrosis factor alpha is a key regulatory molecule for OC maturation 11 and is further important for recruitment of mesenchymal stem cells (MSC) and plays a crucial role in the apoptosis of hypertrophic chondrocytes during endochondral fracture repair 12. Furthermore, bone remodelling is regulated by transforming growth factor beta 1 (TGF‐β1) which stimulates proliferation and differentiation of mesenchymal precursor cells 13 and enhances OC forming potential and survival of OC precursors 14.

The described molecular mechanisms play, at least in part, essential roles during fracture healing and have to be tightly regulated. Related to bone resorption, bone healing in a mouse tibiae fracture model is accompanied by enhanced RANKL, M‐CSF and OPG which are maximally induced within 24 hrs after fracture 15. In addition, M‐CSF and RANKL expression were found to be elevated a second time during endochondral tissue resorption accompanied by increased OC numbers, whereas OPG was relatively decreased. Functional bone regeneration further depends on canonical Wnt signalling, as blockage results in delayed bone fracture healing because of impaired osteoprogenitor cell differentiation 16. Canonical Wnt3a signalling *via* the receptor complex Frizzled and LRP5/6 which led to the accumulation and translocation of β‐catenin into the nucleus is essential for bone formation 17, 18, 19. In addition, non‐canonical Wnt5a signalling acts *via* Frizzled and its co‐receptor Ror2 and Ca^2+^‐dependent enzymes, *e.g*. Ca^2+^ calmodulin‐dependent kinase, or small G proteins or c‐Jun N‐terminal kinase (Jnk) 20 which is involved in bone formation 21 as well as bone resorption 22.

The underlying molecular mechanisms leading to failure of bone regeneration have not been investigated in detail. One key regulator of bone regeneration is bone morphogenetic protein‐2 (BMP‐2) which plays an initial role in bone repair 23. However, it remains unclear whether a dysregulation of BMPs or inhibitors is the reason for regeneration failure. Furthermore, the presence of osteoprogenitor cells in non‐unions and failure of bone regeneration suggest that osteoprogenitor cell differentiation is inhibited 24. Osteoclastogenesis might also be altered during non‐union development as bioinformatical analyses of regular union and non‐union human skeletal fracture microarray data revealed that genes involved in osteclastogenesis are differentially regulated 25. In a study comparing serum levels of patients with long bone atrophic non‐unions and matched control patients OPG serum levels were significantly higher in non‐unions patients, albeit the inability of OPG to inhibit osteoclastic activity is unknown 26.

Thus, the molecular pathology of non‐unions in general is still poorly defined. Our extensive analysis focuses on the late events of scaphoid non‐unions including osteogenesis and osteoclastogenesis, angiogenesis as well as immune response‐related genes compared to cancellous bone from the radius controls in a large cohort, excluding interindividual differences. Our results indicate chronic OC activation in non‐unions, potentially as a result of the altered regulation of *WNT5A* and *TGF‐*β expression which may inhibit bone regeneration, whereas angiogenesis seems to be unaltered in non‐unions.

## Materials and methods

### Human specimens

Tissue harvest and experiments were performed in accordance with the ethical committees, and informed consent was obtained from the patients. Patients with scaphoid non‐unions defined as non‐unified fractures >3 months with a resorption zone wider than 1 mm (as determined by a mandatory CT‐scan) with no apparent potential to heal without surgical intervention were selected to participate in the study. In total, 80 patients from two regional hand trauma centres were recruited and the tissue was processed for RNA and/or histology and intraindividually compared. Non‐union tissue (excluding the cortex) and cancellous bone from the ipsilateral radius has been obtained at the time of operative repair. Patients with previous surgeries on the same scaphoid or conservative treatments were excluded from the study. Seventy‐seven patients were male, three were female. The average age of the patients was 24.6 years (range between 18 and 71 years). The average time that elapsed between fracture and operation was 18.3 months (range 3–100 months).

### Tissue processing

After removal, tissue was immediately washed in ice‐cold PBS to avoid contaminations from blood cells and either frozen at −80°C until RNA preparation or directly processed for histology.

### RNA preparation and cDNA synthesis

Homogenization of the tissue was achieved with Polytron^®^ homogenizer (Kinematica, Eschbach, Germany) in 1 ml TRIzol reagent (Life Technologies, Darmstadt, Germany) on ice. Subsequently, homogenates were incubated at room temperature for 5 min. and 200 μl chloroform (Merck, Darmstadt, Germany) was added and mixed for 5 sec. Samples were centrifuged at 15.300 g for 15 min. at 4°C. The aqueous phase was proceeded for RNA isolation and 1 μl glycogen (Roche, Mannheim, Germany) was added as a carrier. About 250 μl of 100% isopropanol was added and incubated at −80°C over night. After centrifugation at 12,000 r.p.m. for 30 min. at 4°C, samples were incubated and supernatants were removed. Pellets were washed with 1 ml 75% ethanol, centrifuged at 12,000 r.p.m. for 5 min. at 4°C and air‐dried for 20 min. RNA was resuspended in 100 μl RNase‐free water and incubated at 60°C for 10 min. Subsequently, RNA clean up was performed with RNeasy Mini Kit (Qiagen, Hilden, Germany) according to manufacturer's instructions including DNase digestion (RNase free DNase Kit; Qiagen) to avoid genomic DNA contaminations. To limit heterogeneity in the patient population, only young male patients (between 18 and 33 years old) were included. Moreover, only those patients that had high quality RNA (260/280 >1.8, 17 in total) in both tissue samples were included for qRT‐PCR analysis. Synthesis of cDNA was performed by means of the High Capacity cDNA Reverse Transcription Kit with RNase inhibitor (Life Technologies) using 200 ng total RNA per reaction.

### Quantitative real‐time PCR

Quantitative determination of relative gene expression was performed on Applied Biosystems 7900HT Fast Real‐Time PCR System (384 well plates) using TaqMan^®^ gene expression assays (genes and assay IDs are listed in Table 1) and TaqMan^®^ universal master mix (Applied Biosystems, Darmstadt, Germany). For each reaction, 2 ng cDNA were used. Data were analysed according to the manufacturer's ΔΔC_t_ method (Applied Biosystems). 18S was used as a reference gene. Each non‐union sample was related to the corresponding cancellous bone sample control.

**Table 1:** List of genes and TaqMan^®^ gene expression assay IDs examined in the study

*18S*	Hs99999901_s1	*MMP9*	Hs00234579_m1
*ALP*	Hs01029144_m1	*NFATc1*	Hs00542678_m1
*ATF4*	Hs00909569_g1	*NFKB1*	Hs00765730_m1
*BGLAP/OCN*	Hs01587814_g1	*Noggin*	Hs00271352_s1
*BMP2*	Hs00154192_m1	*Osterix/SP7*	Hs01866874_s1
*BMP7*	Hs00233476_m1	*PECAM1*	Hs00169777_m1
*CCND1*	Hs00765553_m1	*RUNX2*	Hs00231692_m1
*CSF1*	Hs00174164_m1	*SPP1/OPN*	Hs00959010_m1
*DKK1*	Hs00183740_m1	*TGFb1*	Hs00998133_m1
*FGF18*	Hs00826077_m1	*TNF‐*α	Hs01113624_g1
*FGF2*	Hs00266645_m1	*TNFRSF11A/RANK*	Hs00921372_m1
*FGF9*	Hs00181829_m1	*TNFRSF11B/OPG*	Hs00900358_m1
*IFNG*	Hs00989291_m1	*TNFSF11/RANKL*	Hs00243522_m1
*IL1B*	Hs01555410_m1	*Wnt3a*	Hs00263977_m1
*MMP13*	Hs00233992_m1	*Wnt5a*	Hs00998537_m1

### Histology and immunohistochemical staining

For histological analyses, tissue was shortly washed with cold PBS to get rid of blood cells, fixed in 4% paraformaldehyde (Sigma‐Aldrich, St. Louis, MO, USA) overnight at 4°C and decalcified in 19% ethylenediaminetetraacetic acid (Applichem, Darmstadt, Germany) for 7 days. Thirty‐four patients were analysed. Afterwards, samples were dehydrated and embedded in paraffin. Bone tissue was cut into serial sections (thickness 9 μm). Pentachrome staining was performed as previously described 27. Tartrate‐resistant acid phosphatase (TRAP) staining was performed with a leucocyte acid phosphatase kit (Sigma‐Aldrich). Immunohistochemistry for PECAM‐1 (#IS610; Dako, Hamburg, Germany) to evaluate blood vessels within the tissue was performed with heat antigen retrieval in citrate buffer (pH 6.0) as previously described 28. Immunohistochemistry for alkaline phosphatase (ALP; #sc166261; Santa Cruz Biotechnology, Heidelberg, Germany, dilution 1:50) was performed after antigen retrieval with 0.1% proteinase K, followed by incubation with antimouse secondary antibody and detection with Vector ABC kit and Nova Red. Immunofluorescence for phosphorylated SMAD2/3 (#8828; Cell Signaling, Frankfurt a. M., Germany, dilution 1:200) was performed overnight at 4°C after antigen retrieval with proteinase K followed by incubation with anti‐rabbit Alexa Fluor 594 secondary antibody (Life Technologies) for 2 hrs at RT and DAPI counterstaining. Immunohistochemistry was performed on samples from at least 13 patients.

### Statistics

Results of qRT‐PCR experiments are presented as mean ± S.E.M. *P* values were calculated with Wilcoxon signed rank test and statistical significances were set at a *P* < 0.05.

## Results

### Regulation of osteogenesis‐related genes

**Figure 1:**
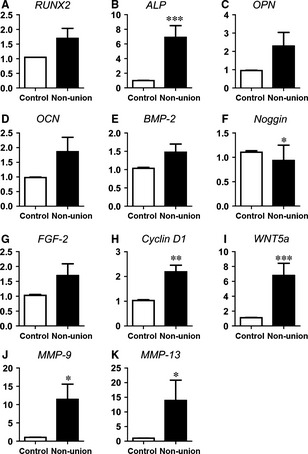
Regulation of osteogenesis‐related genes in human scaphoid non‐unions compared to control adjacent cancellous bone. Quantitative RT‐PCR determination of osteogenesis‐related genes revealed that the majority of genes were significantly up‐regulated (*ALP* (**B**); *cyclin D1* (**H**); *WNT5A* (**I**); *MMP‐9* (**J**); *MMP‐13* (**K**)) compared to controls. Expression levels of *RUNX‐2* (**A**), *OPN* (**C**), *OCN* (**D**), BMP‐2 (**E**) and FGF‐2 (**G**) were similar in both tissues. The BMP inhibitor *noggin* was significantly reduced in non‐unions (**F**). Gene expression determinations were performed relative to 18S expression and data are presented as mean ± S.E.M.; Wilcoxon signed rank test (*P < 0.05, **P < 0.01, ***P < 0.001).

Scaphoid non‐unions are a common problem encountered in clinical practice 29; however, the underlying molecular mechanisms are still poorly defined. To gain insight into the gene expression profiles of bone remodelling and immune response‐related genes of scaphoid non‐unions in comparison to adjacent healthy cancellous bone we performed qRT‐PCR analyses. Osteogenesis and osteoclastogenesis regulating genes as well as pro‐ and anti‐inflammatory markers were included. In scaphoid non‐unions, *RUNX‐2* which is a key transcription factor regulating osteoblastic differentiation, showed similar expression levels compared to control cancellous bone (Fig. 1A). The zinc‐finger containing transcription factor *osterix* known to act downstream of *RUNX‐2* which is essential for bone development 30, was hardly detectable in both tissues (data not shown). Interestingly, OB differentiation marker *ALP* was significantly up‐regulated across all non‐unions (Fig. 1B). In contrast, late OB differentiation markers *osteopontin* (*OPN*) and *osteocalcin* (OCN) showed similar expression patterns in both tissues (Fig. 1C and D). Expression of *BMP‐2* in non‐unions was not differentially regulated as compared to cancellous bone (Fig. 1E). Interestingly, the BMP antagonist *noggin* was moderate, but down‐regulated across all analysed non‐unions except for two patients resulting in overall significant different gene expression (Fig. 1F). In contrast, *BMP‐7* as well as pro‐osteogenic fibroblast growth factors *FGF‐9* and *FGF‐18* 31, 32 were neither detectable in non‐unions nor in control cancellous bone (data not shown). *FGF‐2,* essential for OB proliferation and function, 33 was not differentially expressed (Fig. 1G). *Cyclin D1* required for cell cycle progression 34 was found to be significantly up‐regulated in non‐unions (Fig. 1H). *WNT3A* expression was not detectable in both tissues (data not shown). Interestingly, *WNT5A*, which could interact with canonical Wnt3a pathway, was up‐regulated in non‐unions (mean: 6.7‐fold) (Fig. 1I). Moreover, expression of the matrix metalloproteinases *MMP‐9* and *MMP‐13,* genes related to both angiogenesis and bone remodelling, were investigated. Both, *MMP‐9* (Fig. 1J) as well as *MMP‐13* (Fig. 1K) expression were found to be significantly up‐regulated in non‐unions.

### Osteoclastogenesis‐ and immune response‐related genes

**Figure 2:**
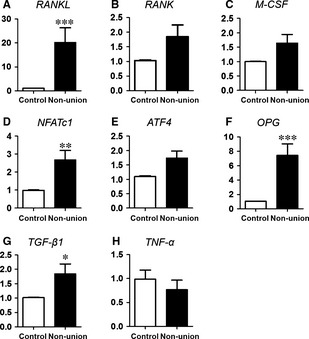
Osteoclastogenesis‐ and immune response‐related genes expressed in human scaphoid non‐unions in comparison to adjacent cancellous bone. Quantitative RT‐PCR determination of *RANKL* (**A**), *NFATc1* (**D**) and *TGF‐*β (**G**) revealed significantly increased expression in scaphoid non‐unions, which are known to have impact on osteoclastogenesis and immune response. Gene expression of *RANK* (**B**), *M‐CSF* (**C**) and *ATF‐4* (**E**) were only slightly increased in non‐unions. Expression analysis of *OPG* (**F**) revealed enhanced expression of the *RANKL* antagonist. Gene expression determinations were performed relative to 18S expression and data are presented as mean ± S.E.M.; Wilcoxon signed rank test (*P < 0.05, **P < 0.01, ***P < 0.001).

Osteoclastogenesis is primarily activated by *RANKL* which regulates OC differentiation processes by induction of transcription factor *NFATc1* and *M‐CSF* known to promote proliferation of monocytic precursor cells. We were interested whether these key molecules of osteoclastogenesis are differentially regulated in non‐unions in comparison to cancellous bone. Quantitative RT‐PCR analysis revealed that *RANKL* was significantly up‐regulated in scaphoid non‐unions (mean: 20‐fold; Fig. 2A). Importantly, *RANKL* expression was significantly up‐regulated in all samples regardless of the time elapsed between trauma and surgery. The RANKL receptor *RANK* was slightly but not significantly up‐regulated in non‐unions compared to controls (Fig. 2B). *M‐CSF* was found to be moderately, but not significantly induced in non‐unions (Fig. 2C) which is because of the high variance of the patient's gene expression. *NFATc1,* a downstream effector, was up‐regulated in non‐unions (Fig. 2D). Recently, *ATF4* was identified as an upstream activator of *NFATc1*. Moreover, *ATF4* is critical for *RANKL* activation and a crucial factor for *M‐CSF* induction of *RANK* expression 35. However, in non‐unions the expression level of *ATF4* was unaltered (Fig. 2E). Interestingly, expression of the soluble decoy receptor of RANKL *OPG* was likewise significantly up‐regulated (Fig. 2F).

Transforming growth factor β was shown to maintain and enhance the OC‐forming potential of OC precursors 14. In scaphoid non‐unions, *TGF‐*β*1* was significantly up‐regulated compared to cancellous bone (Fig. 2G). *NF*κ*B,* which can be induced by *TNF‐*α*,* was neither detected in non‐union nor in cancellous bone (data not shown) which could be because of low *TNF‐*α expression levels which were similar in both tissues (Fig. 2H). Moreover, other pro‐inflammatory cytokines such as *interleukin‐1* (*IL‐1*) and *interferon*‐γ (*IFN‐*γ) were not detected in both tissues.

All results revealed by means of quantitative RT‐PCR analysis are summarized in Table 2.

**Table 2:** Summary of results. Gene expression up‐ and down‐regulation in scaphoid non‐unions compared to control is indicated

*ALP*	↑***	*NFATc1*	↑**
*ATF4*	n.s.	*NFKB1*	n.d.
*BGLAP/OCN*	n.s.	*Noggin*	↓*
*BMP2*	n.s.	*Osterix/SP7*	n.d.
*BMP7*	n.d.	*PECAM1*	n.s.
*CCND1*	↑**	*RUNX2*	n.s.
*CSF1*	n.s.	*SPP1/OPN*	n.s.
*DKK1*	n.s.	*TGFb1*	↑*
*FGF18*	n.d.	*TNF‐*α	n.s.
*FGF2*	n.s.	*TNFRSF11A/RANK*	n.s.
*FGF9*	n.d.	*TNFRSF11B/OPG*	↑***
*IFNG*	n.d.	*TNFSF11/RANKL*	↑***
*IL1B*	n.d.	*Wnt3a*	n.d.
*MMP13*	↑*	*Wnt5a*	↑***
*MMP9*	↑*		

*P < 0.05, **P < 0.01, ***P < 0.001.

n.s.: not significant; n.d.: not detectable.

### Altered architecture, bone remodelling and TGF‐β signalling of scaphoid non‐unions

**Figure 3:**
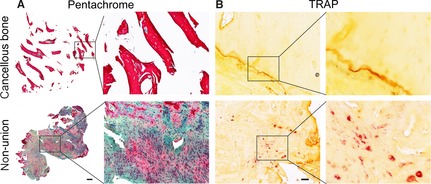
Architecture of scaphoid non‐unions and osteoclast activity. Pentachrome staining revealed a heterogeneous mix of different tissues, with a domination of connective tissue and fibroblasts in non‐unions, while osteoid was the dominant tissue in cancellous bone (**A**). Representative TRAP staining of control cancellous bone and scaphoid non‐unions (**B**) revealed enhanced osteoclasts activity in non‐unions. Scale bars: 200 μm (**A**) and 50 μm (**B**).

**Figure 4:**
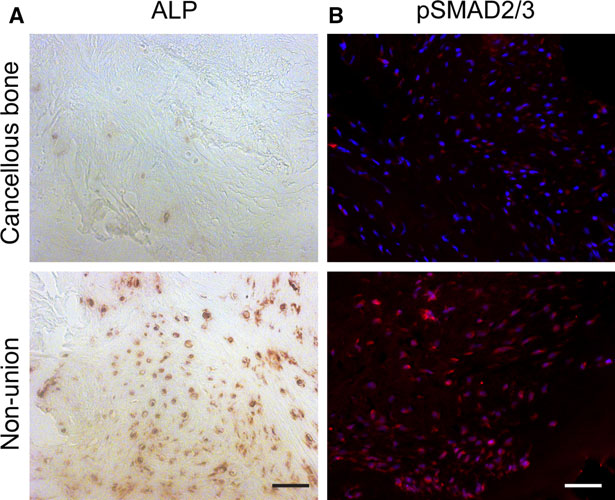
ALP and pSMAD2/3 activity. (**A**) Immunohistochemistry for ALP revealed higher levels in scaphoid non‐unions as opposed to cancellous bone. Likewise, immunofluorescence for phosphorylated SMAD2/3 revealed increased activity in scaphoid non‐unions. Scale bars (**A** and **B**): 50 μm.

Pentachrome staining revealed marked differences between scaphoid non‐unions and healthy cancellous bone tissue. Non‐unions exhibited a heterogeneous mix of different tissues, with a domination of connective tissue, whereas osteoid was the dominant tissue in cancellous bone (Fig. 3A). Gene expression data were supplemented with histological analysis of TRAP‐positive OCs. Comparison of scaphoid non‐unions with control tissue revealed high levels of TRAP staining in non‐unions, indicating increased activity of OCs and confirming gene expression data (Fig. 3B). Of note, OCs were mainly localized in the areas of connective tissue. Moreover, immunohistochemical staining of ALP showed increased activity in scaphoid non‐unions (Fig. 4A) mirroring results obtained in the qRT‐PCR analysis (Fig. 1B) and emphasizing the remaining osteogenic potential of scaphoid non‐unions. Immunohistochemical staining of pSMAD 2/3, a downstream effector of TGF‐β1 revealed highly increased levels in scaphoid non‐unions as compared to control tissue, which further highlights the potential role of TGF‐β1 signalling in scaphoid non‐unions (Fig. 4B).

### Angiogenesis is unaltered in atrophic scaphoid non‐unions

**Figure 5:**
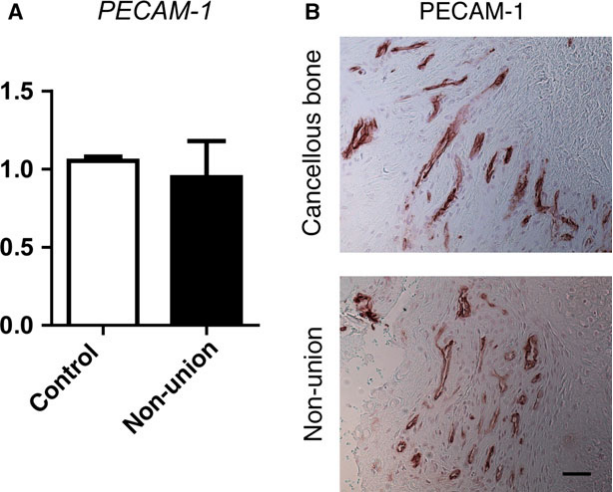
Angiogenesis in scaphoid non‐unions is similar to cancellous bone. (**A**) Quantification of gene expression levels of *PECAM‐1* by qRT‐PCR revealed similar expression levels in non‐unions and controls. Blood vessels and endothelial cells were detected by immunohistochemical staining of PECAM‐1 in non‐unions and controls (**B**) revealing similar levels of angiogenesis in both tissues; scale bar: 50 μm.

As angiogenesis is important for bone development and repair, we compared gene expression of *PECAM‐1* in non‐unions and cancellous bone revealing equal expression levels (Fig. 5A). Concordantly, immunohistochemical staining of PECAM‐1 did not reveal differences between non‐unions and control tissue (Fig. 5B).

## Discussion

Regular fracture healing has been extensively studied but causes for non‐union formation still remain to be elucidated. Here, we performed histological and gene expression analysis for osteogenesis, osteoclastogenesis and immune‐related genes in human atrophic scaphoid non‐unions compared to adjacent cancellous bone. High expression levels of *TGF‐*β, *RANKL* and *NFATc1* as well as increased TRAP‐positive OCs indicate that although trauma may have occurred more than a year before, osteoclastogenesis is constantly induced in non‐unions. Furthermore, our results revealed that non‐unions still have at least a partial regenerative capacity, which seems to be inhibited by increased OC activity.

The structure of non‐unions and cancellous bone is markedly different revealed by pentachrome stainings indicating a dense connective tissue in non‐unions which mainly consists of fibroblasts which is in strong agreement with previous studies 24. As MSC differentiate along osteoblastic and chondrocytic as well as fibroblastic lineages, we speculate that immediately after fracture, MSC differentiation is mainly directed towards the fibroblasts lineage. Initial inflammation after fracture leads to the invasion of macrophages and platelets thereby releasing TGF‐β. Transforming growth factor β was described to enhance fibroblast migration and proliferation in different contexts (reviewed in 36) acquiring an activated phenotype 37. Furthermore, enhanced expression of TGF‐β may lead to sustained fibroblast differentiation and dense persisting fibrous tissue in the fracture gap in an autocrine manner 38, 39. In addition, enhanced TGF‐β expression suggests increased OC survival and differentiation 14. On the contrary, TGF‐β co‐ordinates bone formation by inducing migration of MSC 40 indicating that levels of TGF‐β have to be precisely regulated during bone regeneration. Interestingly, a sheep femoral non‐union model treated with bone allografting indicated that increased numbers of OCs as well as fibroblasts and connective tissue were associated with failure of bone regeneration 36, 41. Our study lets us suggest, that failure of bone regeneration in general is accompanied by connective tissue formation and fibroblast invasion as well as increased OC differentiation as a result of altered TGF‐β and increased phosphorylated SMAD expression.

A key role in the failure of bone regeneration could be increased expression of *RANKL*, its receptor *RANK* and *NFAT1c*, accompanied by OC invasion as indicated by TRAP staining, which could manifest an imbalance of bone formation and bone resorption. Moreover, comparable to a previous study showing elevated OPG serum levels in patients with long bone atrophic non‐union fractures 26, *OPG* expression was highly up‐regulated in scaphoid non‐unions potentially indicating an intact negative feedback loop in response to increased RANKL activity. Furthermore, MMP9 and MMP13 which are important for vascularization, turnover of mineralized cartilage 42 as well as degradation of extracellular matrix in inflammatory responses showed elevated expression levels in scaphoid non‐unions indicating that bone remodelling could occur, but MMPs may also lead to imbalance towards bone resorption.

Interestingly, our experiments revealed that *WNT5a* expression is up‐regulated in scaphoid non‐unions compared to adjacent cancellous bone. In rodents, it was demonstrated that during normal fracture healing Wnt5a is up‐regulated at early stages and down‐regulated to basal levels at later stages of bone healing as compared to non‐injured contralateral tissue 43. Our study revealed that even in long‐term scaphoid non‐unions *WNT5A* gene expression is highly up‐regulated. As *WNT5A* has been shown to indirectly induce RANK expression in OCs thereby enhancing RANKL‐induced osteoclastogenesis, it has been proposed that WNT5a is a new co‐stimulatory cytokine for osteoclastogenesis 22 indicating that increased *RANK* gene expression in scaphoid non‐unions could also result from increased WNT5a expression. On the other hand, WNT5a is up‐regulated during osteoblastic differentiation of MSC thereby regulating expression of RUNX‐2, osterix and ALP 44. In that respect, up‐regulation of ALP in scaphoid non‐unions may be a consequence of increased WNT5A activation, suggesting a certain amount of differentiation capacity of OB progenitor cells. However, as *RUNX‐2* was not differentially regulated, we speculate that WNT5A rather induces osteoclastogenesis than OB differentiation. In another context, WNT5A was shown to stimulate fibroblasts 45 and could be induced by TGF‐β 46, 47 which led us to speculate that *WNT5A* expression is at least partially induced by enhanced *TGF‐*β*1* expression which could lead to fibroblast proliferation and activation. Thus, to this date the exact role of up‐regulated *WNT5A* in established human scaphoid non‐unions is unclear, however, in the light of our results, we speculate that activation of *RANKL* and induction of *WNT5A* expression seems to be one major route of action.

In contrast to *WNT5A*, we neither detected *WNT3A* in scaphoid non‐unions nor in cancellous bone demonstrating that *WNT3A* plays a minor role in established scaphoid non‐unions which does not exclude a role at the early beginning of non‐union development. In addition, *DKK1,* a Wnt antagonist known to inhibit fracture healing 48 is not differentially regulated in late non‐unions, indicating that bone regeneration is not inhibited by DKK1. Interestingly, low levels of β‐catenin as a downstream effector of Wnt3a lead to enhanced OC differentiation and cause osteoporosis 49. Thus, the absence of *WNT3A* could further enhance osteoclastogenesis in scaphoid non‐unions.

We further investigated inflammation‐related which had low expression levels or were neither detectable in non‐unions nor cancellous bone (IL‐1β, IFN‐γ) or were not differentially expressed in non‐unions compared to control tissue (TNF‐α) indicating that local chronic inflammation is presumably not the reason for bone healing failure.

Our results further revealed that angiogenesis was not impaired in non‐unions as *PECAM‐1* gene expression as well as blood vessel numbers were similar compared to control tissue which is in agreement with some previous experimental and non‐comparative data 50, 51, 52. For instance, in a rat model of atrophic non‐union, blood vessel formation was found to be delayed but reaches the same level at later time‐points 51.

**Figure 6:**
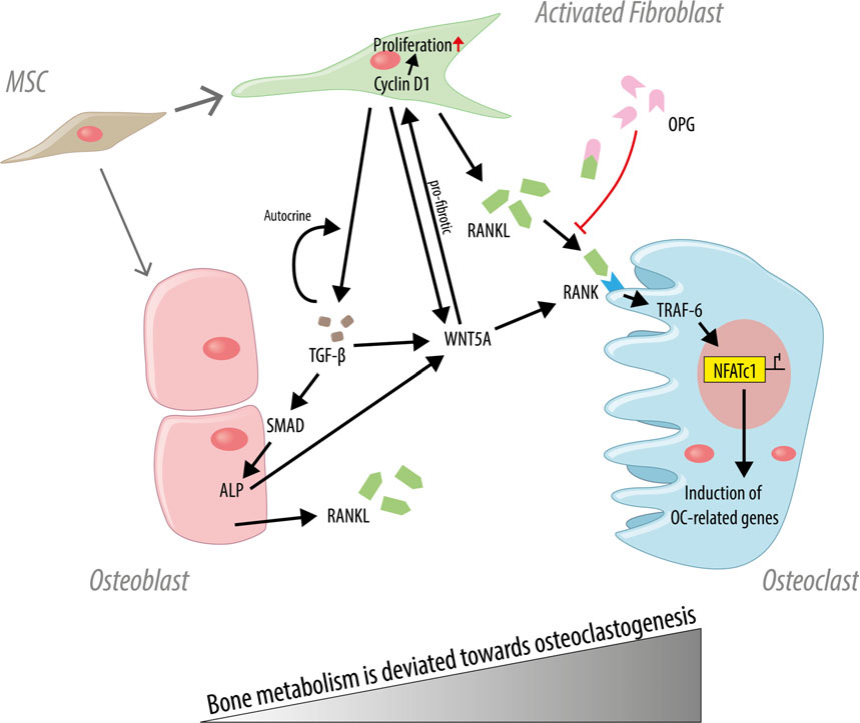
Schematic illustration of the results and hypothesis presented in this study. Autocrine transforming growth factor beta (TGF‐β) signalling leads to activated fibroblasts which express high amounts of receptor activator of nuclear factor‐κB ligand (RANKL) resulting in increased osteoclastogenesis. WNT5A may act pro‐fibrotic or be secreted by osteoblasts (OBs) and fibroblasts and indirectly contribute to osteoclast (OC) differentiation. Differentiation of MSC into OBs is decreased in non‐unions but partial osteogenic differentiation potential of non‐unions still persists as indicated by up‐regulation of alkaline phosphatase (ALP).

In contrast to osteoclastogenesis‐related genes, osteogenesis‐related genes were moderately but not significantly up‐regulated. Although other studies compared BMP‐2 expression levels to regular facture healing showing down‐regulation of BMP‐2 53, 54, comparison of BMP‐2 expression to healthy cancellous bone revealed no significant difference. Hence, detection of BMPs in non‐unions depends on timing of the analysis, location, type of the defect 24 as well as on type of control tissue. Noggin directly binds BMPs which prevents interaction with their receptors resulting in inhibition of BMP signalling 55. Noggin expression was significantly down‐regulated in scaphoid non‐unions compared to the healthy bone which suggests that noggin does not inhibit BMP signalling. Of note, Qu and von Schroeder demonstrated that the addition of recombinant BMPs increases the osteogenic potential of human scaphoid non‐union cells in comparison to pelvic bone cultures 56 indicating at least a partial osteogenic differentiation potential of non‐unions. Our data indeed indicate remaining osteogenic potential in scaphoid non‐unions, which could possibly be exploited, in case the altered balance is readjusted. As studies with human materials are restricted to end‐point analyses, conclusion related to the dynamics of scaphoid non‐union formation are limited. For obvious ethical reasons, the ideal control, contralateral scaphoid cancellous bone, cannot be utilized. However, comparison of non‐union tissue with adjacent cancellous bone excluded interindividual differences. The results revealed by the presented study are summarized in Figure 6.

## Conclusions

In this study, we revealed an imbalance between bone formation and resorption in scaphoid non‐unions. Non‐unions show abnormally high amounts of connective tissue which could result from altered TGF‐β signalling. In an autocrine manner, TGF‐β could further increase fibroblast proliferation and activation. In consequence, fibroblasts express high amounts of RANKL which stimulates osteoclastogenesis. Furthermore, non‐unions showed increased WNT5A expression levels which may also result from altered TGF‐β expression. In addition to TGF‐β blockage and thereby preventing activation of WNT5A, alterations in RANKL and WNT5A expression might also offer therapy approaches. Furthermore, fibroblast proliferation and dense fibrous tissue may be modified. Our data reveal a detailed picture of the status quo of human scaphoid non‐unions and may further accelerate efforts in the field to further understand, prevent and treat this potentially serious musculoskeletal disease.

## Conflicts of interest

The authors confirm that there are no conflicts of interest.

## Author contribution

J.S. analysed quantitative RT‐PCR data including statistics, prepared figures and wrote the manuscript; M.S. contributed to tissue analysis, to tissue collection and preparation; C.D. performed stainings; C.W. prepared figures and contributed to the manuscript; S.A. performed stainings and qRT‐PCR; A.D, U.K and M.L. provided tissue and supervised the study; B.B. designed the research study, analysed stainings and wrote the manuscript.
